# Red (635 nm), Near-Infrared (808 nm) and Violet-Blue (405 nm) Photobiomodulation Potentiality on Human Osteoblasts and Mesenchymal Stromal Cells: A Morphological and Molecular In Vitro Study

**DOI:** 10.3390/ijms19071946

**Published:** 2018-07-03

**Authors:** Alessia Tani, Flaminia Chellini, Marco Giannelli, Daniele Nosi, Sandra Zecchi-Orlandini, Chiara Sassoli

**Affiliations:** 1 Department of Experimental and Clinical Medicine—Section of Anatomy and Histology, University of Florence, Largo Brambilla 3, 50134 Florence, Italy; alessia.tani@unifi.it (A.T.); flaminia.chellini@unifi.it (F.C.); daniele.nosi@unifi.it (D.N.); zecchi@unifi.it (S.Z.-O.); 2 Odontostomatologic Laser Therapy Center, via dell’ Olivuzzo 162, 50143 Florence, Italy; dott.giannellimarco@dada.it

**Keywords:** photobiomodulation (PBM), low level laser therapy (LLLT), diode laser, light emitting diode (LED), osteoblasts, mesenchymal stromal cells, bone regeneration, Runx-2, ostepontin, Akt signaling

## Abstract

Photobiomodulation (PBM) has been used for bone regenerative purposes in different fields of medicine and dentistry, but contradictory results demand a skeptical look for its potential benefits. This in vitro study compared PBM potentiality by red (635 ± 5 nm) or near-infrared (NIR, 808 ± 10 nm) diode lasers and violet-blue (405 ± 5 nm) light-emitting diode operating in a continuous wave with a 0.4 J/cm^2^ energy density, on human osteoblast and mesenchymal stromal cell (hMSC) viability, proliferation, adhesion and osteogenic differentiation. PBM treatments did not alter viability (PI/Syto16 and MTS assays). Confocal immunofluorescence and RT-PCR analyses indicated that red PBM (i) on both cell types increased vinculin-rich clusters, osteogenic markers expression (Runx-2, alkaline phosphatase, osteopontin) and mineralized bone-like nodule structure deposition and (ii) on hMSCs induced stress fiber formation and upregulated the expression of proliferation marker Ki67. Interestingly, osteoblast responses to red light were mediated by Akt signaling activation, which seems to positively modulate reactive oxygen species levels. Violet-blue light-irradiated cells behaved essentially as untreated ones and NIR irradiated ones displayed modifications of cytoskeleton assembly, Runx-2 expression and mineralization pattern. Although within the limitations of an in vitro experimentation, this study may suggest PBM with 635 nm laser as potential effective option for promoting/improving bone regeneration.

## 1. Introduction

Bone tissue defects can derive from many different conditions such as congenital anomalies, trauma or cancer excision and can eventually lead to a significant patient morbidity with a proportional increase in social costs. Bone destruction and loss also represent serious complications of periodontitis and peri-implantitis [1,2] and, in such a perspective, the achievement of satisfactory bone repair/regeneration remains an important goal both in orthopedics and dentistry.

Bone tissue possesses a remarkable capacity of self-repair and regeneration. The bone regenerative process requires the interplay of different cells including inflammatory cells, osteoclasts and osteogenic cells. Among the latter, pre-osteoblasts and osteoblasts are the primarily responsibilities of bone matrix building and new functional tissue formation [3,4]. A crucial role in this process is played by mesenchymal stromal cells (MSCs) which, beside differentiating in bone-forming osteoblasts, may secrete several paracrine factors (growth factors and cytokines) with multiple beneficial effects on the tissue microenvironment, including reduction of inflammation and modulation of the functionality of resident osteogenic cells. These cells may be recruited to the bone injury site from periosteum, endosteum, bone marrow (endogenous MSCs) or from systemic circulation [4,5]. However, the regenerative ability of bone could be hampered by adverse conditions such as pathological fractures or critical-sized bone injuries/defects, with consequent bone-healing failure which can represent a serious challenge to a reconstructive surgeon. Moreover, other pathologies such as insufficient blood supply, osteomyelitis, and systemic diseases (e.g., diabetes) may contribute to limit bone repair/regeneration [6].

Numerous research has been focused in recent decades on the identification of potential strategies mainly aimed at positively influencing the functionality of bone progenitor cells. The encouraging results have been paralleled by advancements in bone tissue engineering [7]. Nevertheless, bone reconstruction still faces many challenges and at present bone tissue autograft or allograft transplantations remain the treatments of choice, even considering the correlated critical issues. Of note, bone represents the second most commonly transplanted tissue after blood [8].

In this scenario, the identification of alternative and effective therapeutical options aimed at promoting bone healing represents a clinical need with a potentially high impact on social wealth.

Low-Level Laser Therapy (LLLT) or photobiomodulation (PBM) has been explored as a promising non-invasive and painless therapeutic strategy to encourage bone regeneration in different fields of medicine and dentistry. It consists in the direct application of coherent (lasers) or non-coherent (light-emitting diode, LED) light sources with a wavelength between 405 and 1100 nm and output power less than hundreds of milliwatts and energy density less than 10 J/cm^2^ [9]. Given these features, the light can easily enter the tissues in a non-destructive mode, with negligible thermal sound or vibration effects, and induce photochemical reactions resulting in the modulation of different cellular processes.

The effectiveness of PBM to encourage proliferation and/or differentiation of osteogenic cells has been described in many in vitro studies [10,11,12,13,14,15,16,17,18,19,20,21]. In addition, several pieces of in vivo research addressed the ability of PBM to accelerate the process of bone healing after oral and orthopedic surgery, by inducing an increase in bone density, neoformation, regeneration and mineralization [22,23,24,25,26,27,28] and some clinical trials are currently ongoing in different centers all over the world (Available online: https://clinicaltrials.gov/). However, at present, univocal standardized guidelines for the use of PBM for osteoregenerative purposes are not available. This is mainly dependent on the variety of wavelengths, medical laser and LED types used with disparate energy output modes and setting parameters, which has produced many different treatment protocols with different and sometimes contradictory outcomes [13,20,21,26,29,30] hampering meaningful comparison of the results and demanding a skeptical look for the potential beneficial effects of this approach. Moreover, this scenario is complicated by the fact that a biphasic-dose cellular response to the same wavelength has been frequently observed with low levels of light that are more effective than the higher ones on stimulating cell functionality [31] In addition, the molecular mechanisms by which PBM induces different biological responses have not been fully clarified. Therefore, optimization of the parameters by identifying the most appropriate and effective wavelengths, beam type, energy density (fluence), irradiation mode as well as exposure time able to positively impact on the osteoprogenitor cell behavior, represents a priority to obtain consistent results allowing the design of effective treatment protocols. Based on these considerations, the purpose of the present in vitro study was to compare the potentiality of three PBM treatments by 635 ± 5 nm (visible red) diode laser or 808 ± 10 nm (near-infrared, NIR) GaAlAs diode laser and 405 ± 5 nm (violet-blue) LED to biostimulate human osteoblasts and bone marrow-derived MSCs (hMSCs) in terms of viability, proliferation, adhesion and osteogenic differentiation. In addition, we investigated the molecular mechanisms underpinning the PBM treatment action.

We tested these wavelengths on the basis of previous reports showing the ability of 635 nm to modulate different cell type behavior [11,32], and the in vitro efficacy of photodynamic treatment with methylene blue photoactivated with 635 nm or with 405 nm wavelengths [33] as well as of 808 nm laser phototreatment [34] to selectively inactivate different bacteria playing a central role in the pathogenesis of periodontal and peri-implant diseases. In addition, the bactericidal effect of 405 nm has also been demonstrated on a variety of pathogenic bacteria related to the infections after orthopedic surgery [35]. Our idea is that the positive influence of these phototreatments also on bone progenitor cells could extend the list of beneficial effects of laser/LED therapy and therefore their application.

The present study demonstrated that among the different tested PBM treatments, the one carried out with 635 nm diode laser at energy density of 0.4 J/cm^2^, is able to positively influence the behavior of both osteoblasts and hMSCs and that the osteoblast responses to the treatment are mediated by Akt signaling activation.

## 2. Results

### 2.1. Human Osteoblast Viability, Proliferation, Adhesion and Differentiation

Saos-2 osteoblasts were exposed to three different treatments of PBM with 635 ± 5 nm, 808 ± 10 nm or 405 nm and assessed for cell viability, proliferation, adhesion and differentiation. Light sources and the irradiation parameters are reported in Table 1.

**Figure 1:**
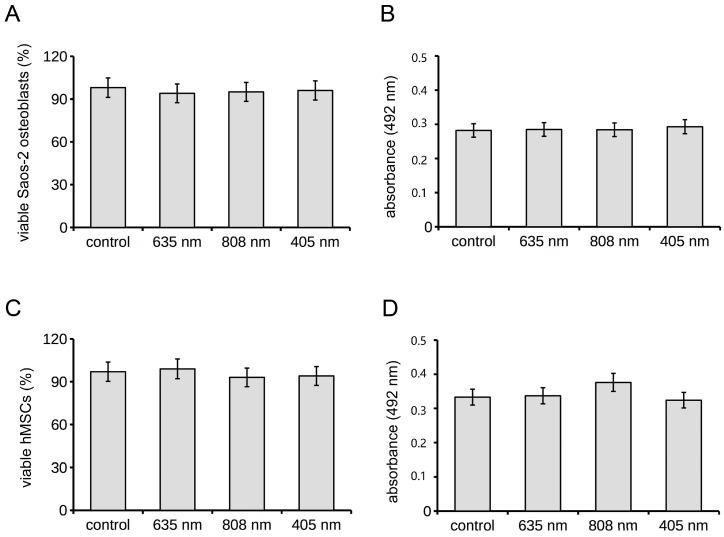
Effects of red (635 nm), NIR (808 nm) and violet-blue (405 nm) PBM on osteoblast and hMSC viability. Osteoblasts and hMSCs subjected or not (control) to PBM treatments with 635 nm, 808 nm or 405 nm as reported in Table 1, were cultured for 24 h in their specific PM and then assessed for cell viability. (**A**,**C**) PI/Syto16 test. Histograms show the morphometric analysis of percentage of Syto16 positive viable (**A**) osteoblasts and (**C**) hMSCs. (**B**,**D**) MTS assay. Absorbance (492 nm) of soluble formazan resulting from the reduction of tetrazolium by mitochondrial enzymes of viable (**B**) osteoblasts and (**D**) hMSCs, measured using a multi-well scanning spectrophotometer.

Untreated cells served as controls. The cells were monitored daily under an inverted phase contrast microscope and neither red, NIR and violet-blue PBM treatments caused cellular morphological changes. The treated cells were mostly negative to nuclear PI staining, a marker of dead cells (Figure 1A), thus excluding any cytotoxic effect by irradiations. Moreover, when assessed by MTS assay after 24 h from light application, no differences in viability of Saos-2 osteoblasts were observed after stimulation with the different PBM treatments as compared to control cells (Figure 1B).

**Figure 2:**
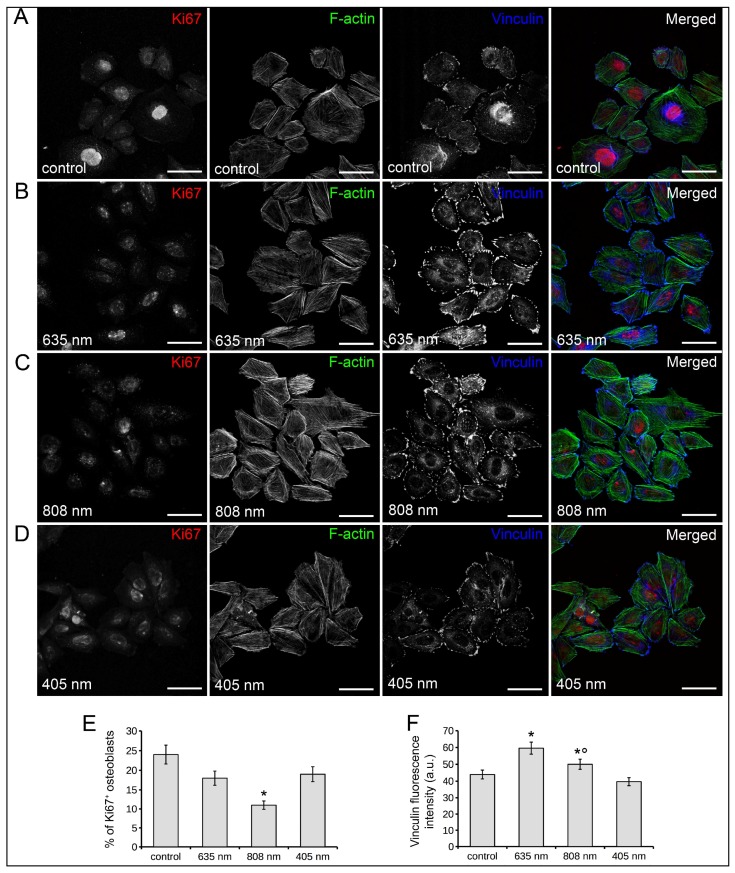
Effects of red (635 nm), NIR (808 nm) and violet-blue (405 nm) on osteoblast proliferation and cytoskeleton assembly. Osteoblasts subjected or not (control) to PBM with 635 nm, 808 nm or 405 nm as reported in Table 1, were cultured for 24 h in PM. (**A**–**D**) Representative confocal fluorescence images of fixed cells in the indicated experimental conditions, immunostained with antibodies against the proliferation marker Ki67 (red) and against the focal adhesion protein vinculin (cyan) and stained with Alexa 488-conjugated phalloidin to reveal F-actin (green). Scale bar: 50 µm (**E**). Quantitative analysis of Ki67 positive osteoblast nuclei expressed as percentage of the total nuclei number. (**F**) Histogram showing the densitometric analysis of the intensity of vinculin fluorescence signal performed on digitized images. The data are representative of at least three independent experiments, performed in triplicate. The values are expressed as mean ± S.E.M. Significance of difference: * *p* < 0.05 vs. control, ° *p* < 0.05 vs. 635 nm.

Confocal immunofluorescence examination for the expression of the nuclear proliferation marker Ki67 showed no significant differences in the percentage of cells positive for this marker at 24 h post phototreatments with 635 nm or 405 nm as compared to control cells (Figure 2A,B,D,E).

By contrast, the proliferative ability of osteoblasts appeared significantly reduced 24 h after PBM with 808 nm (Figure 2C,E). Consistent with these results, 808 nm induced a prominent cytoskeletal rearrangement, with the formation of massive well-defined F-actin filaments possibly stress fibers (Figure 2C) and an increased expression of the focal adhesion protein vinculin aggregated in large clusters at the end of the filaments, a feature typical of substrate-adherent cells (Figure 2C,F) as compared to controls (Figure 2A,F) in which thin F-actin filaments appeared arranged in a web-like structure or in parallel arrays whereas vinculin accumulated in small dot-like aggregates at either the cell border and scattered throughout the cytoplasm. Of note, cells irradiated with 635 nm displayed an actin cytoskeleton assembly comparable to that of control cells but, differently from controls, they displayed an increase of vinculin-rich focal adhesion sites predominantly at the periphery of the cells (Figure 2A,B,F). Cytoskeleton assembly as well as vinculin expression, localization and distribution pattern in osteoblasts exposed to 405 nm were comparable to those of control cells (Figure 2A,D,F).

**Figure 3:**
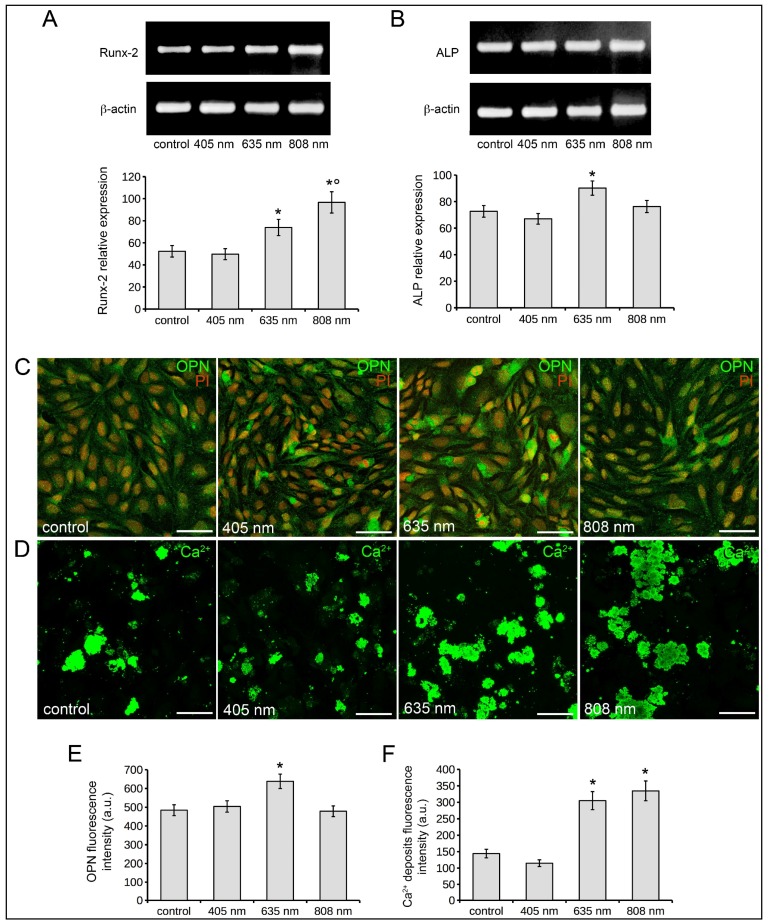
Effects of red (635 nm), NIR (808 nm) and violet-blue (405 nm) PBM on osteoblast differentiation. Osteoblasts subjected or not (control) to PBM treatments with 635 nm, 808 nm or 405 nm as reported in Table 1, were cultured for 7 and 18 days in osteogenic DM. (**A**,**B**) RT-PCR analysis of (**A**) Runx-2 and (**B**) ALP expression in the cells cultured for 7 days in DM in the indicated experimental conditions. Representative agarose gels are shown. The densitometric analyses of the bands normalized to β-actin are reported in the histograms. (**C**,**D**) Representative confocal fluorescence images of cells cultured in DM in the indicated experimental conditions. In (**C**) the cells were cultured for 7 days, fixed and immunostained with antibodies against osteopontin (OPN, green) and stained with PI (red) to reveal nuclei. In (**D**) the cells were cultured for 18 days, fixed and stained with the fluorescent Osteolmage™ staining reagent (green) binding the hydroxyapatite portion of the bone like nodule structures deposited by cells (Ca^2+^ deposits). Scale bar: 50 µm. (**E**,**F**) Histograms showing the densitometric analyses of the intensity of (**E**) OPN and (**F**) Ca^2+^ deposits fluorescence signals performed on digitized images. The data are representative of at least three independent experiments, performed in triplicate. The values are expressed as mean ± S.E.M. Significance of difference: * *p* < 0.05 vs. control, ° *p* < 0.05 vs. 635 nm.

Osteoblast differentiation was assessed by the evaluation and quantification of Runx-2, alkaline phosphatase (ALP), osteopontin (OPN) expression and of Ca^2+^ deposits, assumed as early and late osteoblast differentiation markers, in Saos-2 cells exposed to the three different PBM treatments and cultured in osteogenic differentiation medium (DM) for 7 or 18 days. The relative mRNA expression of Runx-2 and ALP normalized to β-actin, evaluated by RT-PCR analyses, at 7 days after light application, is shown in Figure 3A,B. A statistically significant up-regulation of both Runx-2 (Figure 3A) and ALP (Figure 3B) mRNA expression was observed in osteoblasts after PBM with 635 nm as compared to controls. 808 nm induced an increase of mRNA expression of Runx-2 (Figure 3A) but not of ALP (Figure 3B). No variations in the expression of these genes were detected in the cells subjected to PBM with 405 nm, as compared to control cells.

As judged by confocal immunofluorescence analysis, the expression of OPN, a bone matrix non-collagenous glyco-phosphoprotein secreted by osteoblasts during bone mineralization and remodeling [36], displaying a punctate fluorescence pattern scattered throughout the cytoplasm, resulted in an increase in cells treated with 635 nm, 7 days post phototreatment as compared to controls. OPN expression in osteoblasts irradiated with 808 nm or 405 nm appeared similar to that of control cells (Figure 3C,E). Finally, the fluorescence analysis of mineralized bone-like nodule structures (Ca^2+^ deposits) in osteoblasts 18 days post red and NIR PBM showed a significant increase of these structures as judged by the higher fluorescent signal intensity with respect to control cells. The pattern of mineralization was not significantly altered by violet-blue light (Figure 3D,F).

Altogether these data may suggest that red PBM carried out with 635 nm diode laser promotes osteoblast differentiation.

### 2.2. Human MSC Viability, Proliferation, Adhesion and Osteogenic Differentiation

**Figure 4:**
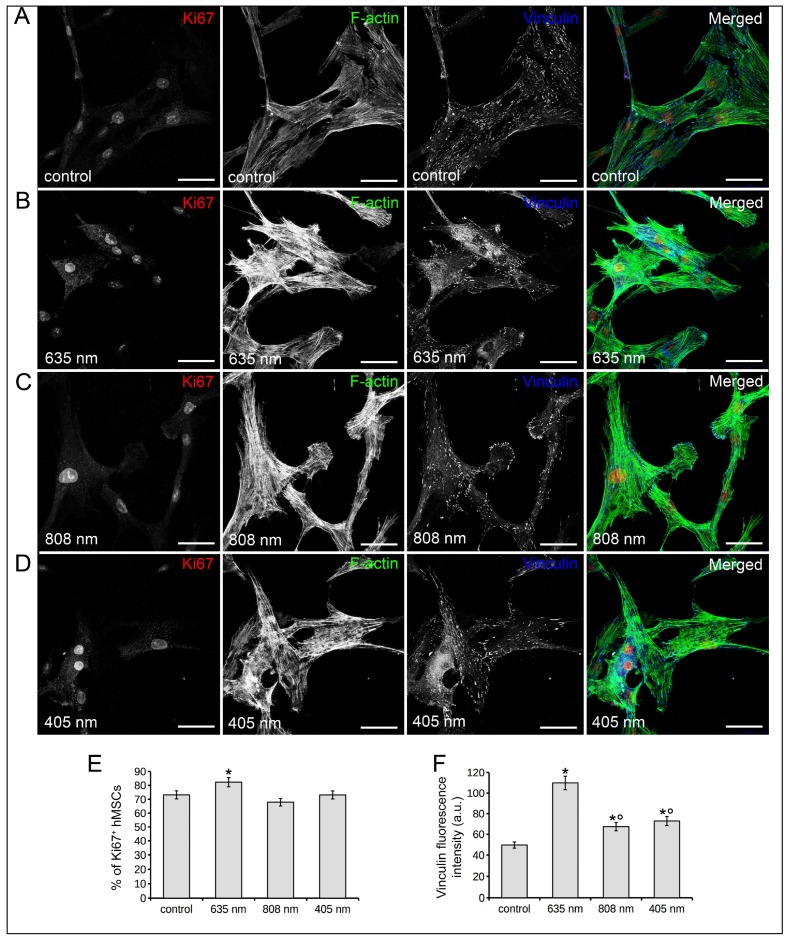
Effects of red (635 nm), NIR (808 nm) and violet-blue (405 nm) PBM on hMSC proliferation and cytoskeleton assembly. hMSCs subjected or not (control) to PBM treatments with 635 nm, 808 nm or 405 nm as reported in Table 1, were cultured for 24 h in PM. (**A**–**D**) Representative confocal fluorescence images of fixed cells in the indicated experimental conditions, immunostained with antibodies against the proliferation marker Ki67 (red) and against the focal adhesion protein vinculin (cyan) and stained with Alexa 488-conjugated phalloidin to reveal F-actin (green). Scale bar: 50 µm. (**E**) Quantitative analysis of Ki67 positive hMSC nuclei expressed as percentage of the total nuclei number. (**F**) Histogram showing the densitometric analysis of the intensity of vinculin fluorescence signal performed on digitized images. The data are representative of at least three independent experiments, performed in triplicate. The values are expressed as mean ± S.E.M. Significance of difference: * *p* < 0.05 vs. control, ° *p* < 0.05 vs. 635 nm.

The viability of human bone marrow-derived MSCs (hMSCs) subjected to the three different red, NIR and violet-blue PBM treatments was comparable to that of untreated cells, as shown by the results of nuclear PI/Syto16 staining test (Figure 1C) and MTS assay (Figure 1D). A slight but significant increase in the percentage of Ki67 positive cells was found when the cells were treated with 635 nm as compared to control cells, in accordance with our previous observations on murine bone marrow-derived MSCs [11] (Figure 4A,B,E). Conversely, 808 nm and 405 nm did not alter the proliferative ability of the cells as compared to controls (Figure 4A,C–E). hMSCs irradiated with the different PBM treatments displayed a more robust well-structured F-actin filaments spanning through the length of the cells and more numerous vinculin-rich focal adhesion sites with respect to untreated cells (Figure 4A–D,F). The highest increase in vinculin-rich plaques was induced by 635 nm.

**Figure 5:**
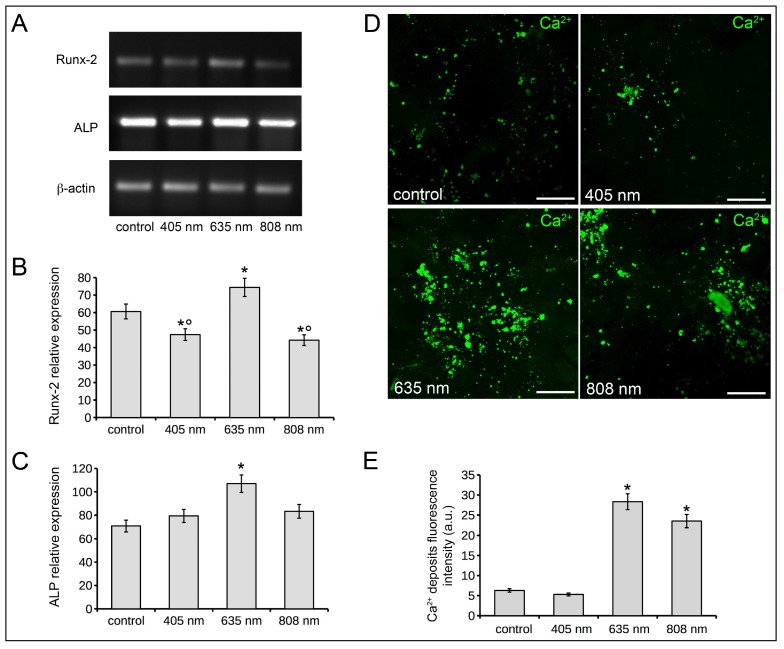
Effects of red, NIR and violet-blue on hMSC osteogenic differentiation. hMSCs subjected or not (control) to PBM treatments with 635 nm, 808 nm or 405 nm as reported in Table 1, were cultured for 7 and 18 days in osteogenic DM. (**A**–**C**) RT-PCR analyses of Runx-2 and ALP expression in the cells cultured for 7 days in DM in the indicated experimental conditions. (**A**) Representative agarose gels are shown. (**B**,**C**) Histograms showing the densitometric analyses of the (**B**) Runx-2 and (**C**) ALP bands normalized to β-actin. (**D**) Representative confocal fluorescence images of cells cultured in DM in the indicated experimental conditions for 18 days, fixed and stained with the fluorescent Osteolmage™ staining reagent (green) binding the hydroxyapatite portion of the bone-like nodule structures deposited by cells (Ca^2+^ deposits). Scale bar: 50 µm. (**E**) Histogram showing the densitometric analysis of the intensity of Ca^2+^ deposits fluorescence signal performed on digitized images. The data are representative of at least three independent experiments, performed in triplicate. The values are expressed as mean ± S.E.M. Significance of difference: * *p* < 0.05 vs. control, ° *p* < 0.05 vs. 635 nm.

The evaluation of hMSC osteogenic differentiation, as assessed by RT-PCR analyses of mRNA expression of Runx-2 and ALP, and by the fluorescence analysis of mineralized bone-like nodule structure formation showed that: (i) 635 nm increased the levels of Runx-2 and ALP mRNA expression in the cells cultured for 7 days in osteogenic DM with respect to control (Figure 5A–C); (ii) a slight but significant reduction of Runx-2 mRNA expression was found in cells subjected to PBM with 808 nm or 405 nm (Figure 5A,B); (iii) no substantial changes in the expression of ALP mRNA were detected in the cells after PBM with 808 nm or 405 nm as compared to controls (Figure 5A,C); (iv) hMSCs cultured for 18 days in osteogenic DM displayed deposition of mineralized bone-like nodule structures (Ca^2+^ deposits) on glass coverslips and Ca^2+^ deposits fluorescent signal intensity in the cells irradiated with red or near- infrared light resulted higher than that related to control cells (Figure 5D,E). Similar to osteoblast response, the pattern of mineralization of hMSCs was not significantly altered by 405 nm (Figure 5D,E). Altogether these data may suggest that among the tested PBM treatments, the red one using 635 nm diode laser is capable of promoting hMSC proliferation, adhesion and osteogenic differentiation.

### 2.3. Osteoblast Responses to PBM with 635 nm are Mediated by Akt Signaling Activation

**Figure 6:**
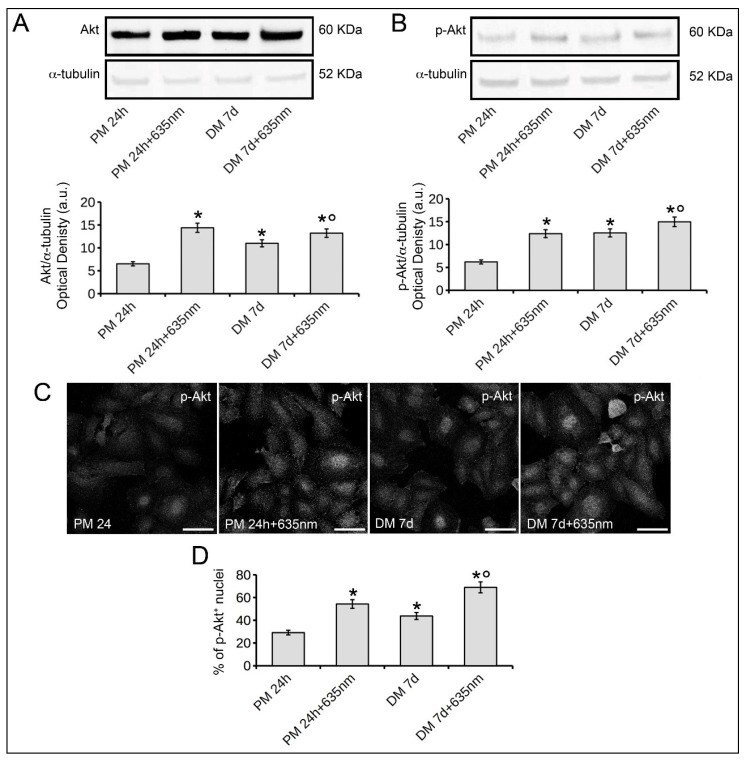
Effects of red PBM on osteoblast Akt expression and activation. Osteoblasts subjected or not to PBM with 635 nm as reported in Table 1, were cultured for 24 h in PM or for 7 days (d) in osteogenic DM. (**A**,**B**) Western blotting analyses of (**A**) Akt and (**B**) the activated phosphorylated form of Akt, p-Akt, expression. The densitometric analyses of the bands normalized to α-tubulin are reported in the histograms. (**C**) Representative confocal immunofluorescence images of fixed cells immunostained with antibodies against p-Akt. Scale bar: 50 µm. (**D**) Quantitative analysis of p-Akt positive osteoblast nuclei expressed as percentage of the total nuclei number. The data are representative of at least three independent experiments, performed in triplicate. The values are expressed as mean ± S.E.M. Significance of difference: * *p* < 0.05 vs. PM 24 h, ° *p* < 0.05 vs. DM 7 days.

Based on the observed effects of the red PBM treatment on osteoblasts, namely increase of the expression and aggregation of vinculin and of the expression of osteogenic differentiation markers, we next performed experiments aimed at investigating the molecular mechanisms underlying the effects of the PBM treatment. In particular, we analyzed the involvement of Akt-mediated signaling. Western blotting analyses revealed the expression of Akt and of its activated phosphorylated form, p-Akt, in osteoblasts cultured in PM for 24 h as well as in DM for 7 days (Figure 6A,B). Of note, the cells irradiated with 635 nm and cultured for 24 h in PM or 7 days in DM showed higher expression levels of both Akt and p-Akt than those of the respective control cells (Figure 6A,B). Confocal immunofluorescence analysis of p-Akt confirmed these data; indeed, the percentage of p-Akt positive osteoblast nuclei was higher in laser treated cells as compared to untreated ones (Figure 6C,D).

**Figure 7:**
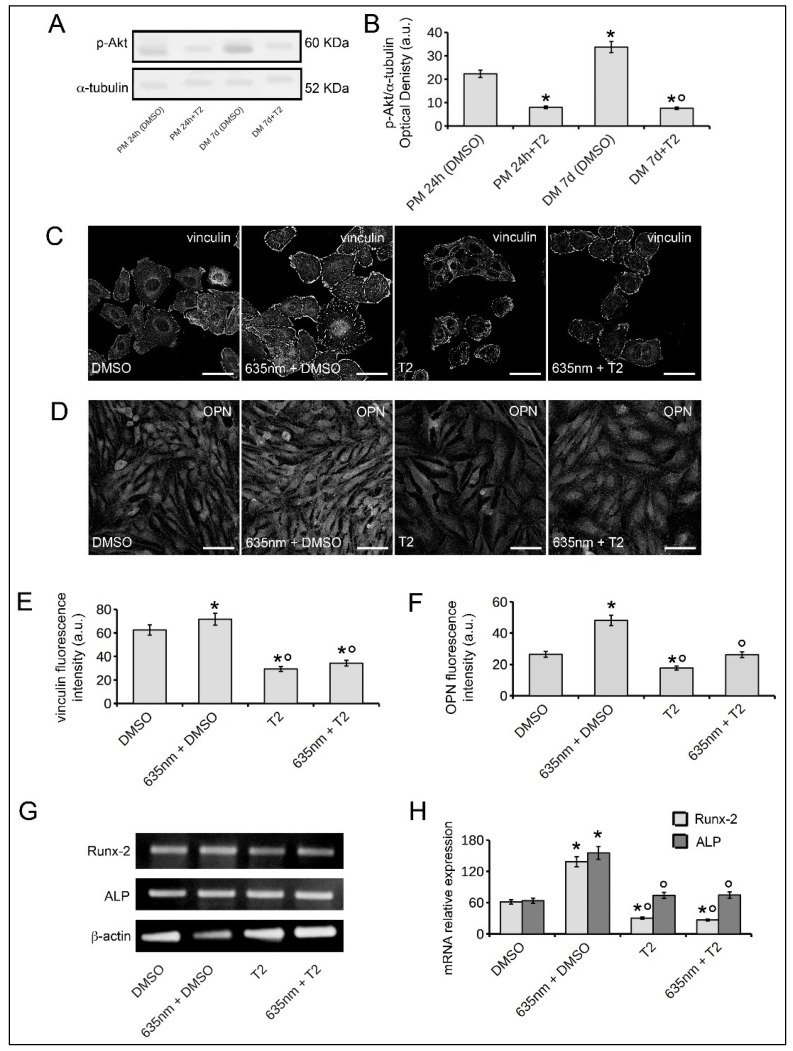
Assessment of Akt signaling involvement in osteoblast responses induced by red PBM. Osteoblasts subjected or not to PBM with 635 nm as reported in Table 1, were cultured for 24 h in PM or for 7 days (d) in osteogenic DM in the absence (DMSO) or presence of the Akt inhibitor, triciribine (2 µM, T2). (**A**) Western blotting analysis of p-Akt expression. (**B**) Histogram showing the densitometric analysis of the bands normalized to α-tubulin. (**C**,**D**) Representative confocal immunofluorescence images of cells cultured in the indicated experimental conditions. In (**C**) the cells were cultured in PM for 24 h, fixed immunostained with antibodies against vinculin; in (**D**) the cells were cultured in DM for 7 days, fixed and stained with antibodies against osteopontin OPN. Scale bar: 50 µm. (**E**,**F**) Histograms showing the densitometric analyses of the intensity of (**E**) vinculin and (**F**) OPN fluorescence signals performed on digitized images. (**G**) RT-PCR analyses of Runx-2 and ALP expression in the cells cultured for 7 days in DM in the indicated experimental conditions. Representative agarose gels are shown. (**H**) Histograms showing the densitometric analyses of Runx-2 and ALP bands normalized to β-actin. The data are representative of at least three independent experiments, performed in triplicate. The values are expressed as mean ± S.E.M. Significance of difference: in (**B**), * *p* < 0.05 vs. PM 24 h (DMSO), ° *p* < 0.05 vs. DM 7 days (DMSO); in (**E**,**F**,**H**), * *p* < 0.05 vs. DMSO, ° *p* < 0.05 vs. 635 nm + DMSO.

To address the involvement of Akt-mediated pathway on red PBM-induced osteoblast responses further, the cells were exposed to laser irradiation in the presence of 2 µM triciribine (T2), an inhibitor of Akt signaling shown to reduce p-Akt expression (Figure 7A,B).

The expression of p-Akt in osteoblasts cultured in the absence or presence of 0.1% dimethyl sulfoxide (DMSO, vehicle) was comparable (data not shown). T2 in our culture condition did not affect cell viability; indeed as judged by PI/Syto16 test, the cells cultured for 24 h in PM or 7 days in DM in the presence of T2 were mostly negative to nuclear PI staining (percentage of viable cells cultured for 24 h in PM: untreated cells, 98 ± 2.3, T2 treated cells, 96 ± 3.1; percentage of viable cells cultured for 7 days in DM: untreated cells 96 ± 4.2, T2 treated cells: 97 ± 3.9; *p* > 0.05).

Notably the blockade of Akt-mediated pathway strongly reduced the effects of 635 nm on vinculin expression (Figure 7C,E), on OPN expression (Figure 7D,F) and on Runx-2 and ALP mRNA expression (Figure 7G,H). No differences in the responses of laser-irradiated osteoblasts in the absence or presence of 0.1% DMSO (vehicle) were found.

**Figure 8:**
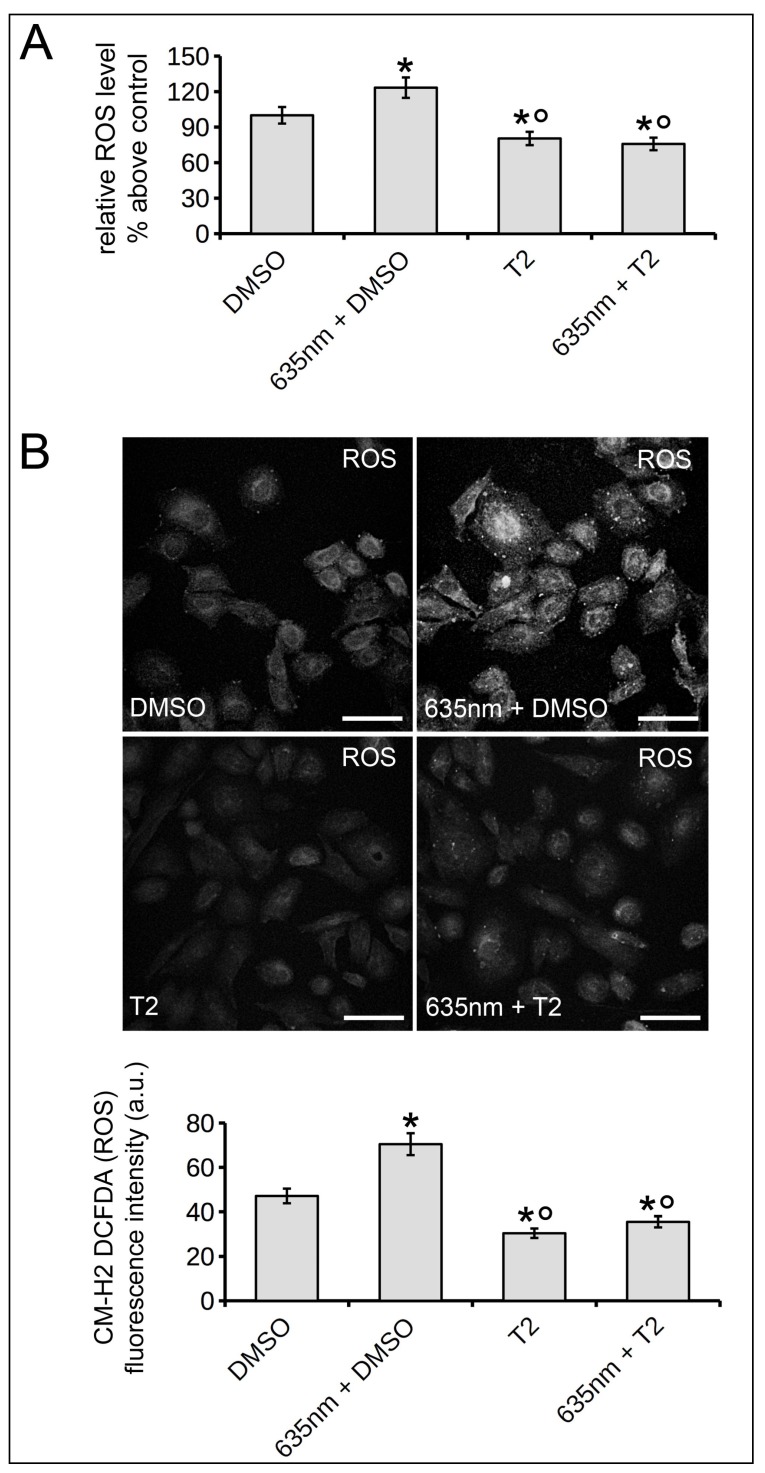
Effect of red PBM and Akt signaling inhibition on ROS production in osteoblasts. ROS generation was measured with CM-H_2_ DCFDA in osteoblasts cultured in the absence (DMSO) or presence of the Akt inhibitor triciribine (2 µM, T2) subjected or not to PBM with 635 nm as reported in Table 1, 1 h after light delivery. (**A**) Fluorometric ROS detection. (**B**) Representative confocal fluorescence images of intracellular ROS. Scale bar: 50 µm. The histogram shows the densitometric analyses of the intensity of CM-H_2_ DCFDA (ROS) green fluorescence signals performed on digitized images. The data are representative of at least three independent experiments, performed in triplicate. The values are expressed as mean ± S.E.M. Significance of difference: * *p* < 0.05 vs. DMSO, ° *p* < 0.05 vs. 635 nm + DMSO.

Finally, we also found that 635 nm induced an increase in intracellular levels of reactive oxygen species (ROS), which was prevented when the red light was applied in the presence of T2 (Figure 8).

Thus, it may be suggested that Akt-mediated signaling pathway, possibly positively modulating ROS levels, is involved in osteoblast responses induced by 635 nm laser irradiation.

## 3. Discussion

This study provides novel experimental evidence of the ability of red PBM treatment carried out with a 635 ± 5 nm diode laser operating in a continuous wave, without contact, with 0.4 J/cm^2^ energy density, to modulate the functionality of different human progenitor cells playing a key role in bone formation and regeneration, such as osteoblasts and MSCs [3,4,5], in terms of proliferation, adhesion and differentiation. Moreover, our data contribute toward defining the molecular mechanisms underpinning the laser action, showing the involvement of Akt signaling in mediating the laser induced osteoblast responses.

Although within the limitations of an in vitro experimentation, this study may suggest red PBM treatment as a potential effective option for promoting or improving bone regeneration, in addition, as comparative analysis among red, NIR and violet-blue PBM effects on bone progenitor cells, it may contribute towards discovering the most suitable wavelengths to be used for this purpose. Indeed, we demonstrated that as compared to 405 nm, 635 nm in osteoblasts induced: (i) an increase of the expression of vinculin, one of the mechanosensing adhesion protein by which osteoblasts sense matrix stiffness, recognized as a critical regulator of different osteoblast processes including growth, migration, differentiation as well as phenotype maintenance [37,38]; and (ii) an upregulation of all tested osteogenic differentiation markers, namely Runx-2, ALP, OPN as well as an increase of mineralized bone-like nodule structure formation. Our results agree with a recent report showing that PBM with 635 nm did not affect viability and proliferation of CRL-11372 osteoblast-like cells [30]. In contrast with our findings, that study showed no significant stimulatory effects of this PBM treatment on osteoblast differentiation; this may be dependent on the different responsiveness of the two osteoblast-like cell types to red light or on the different laser parameters used, such as the higher energy doses (0.5, 1, 2 J/cm^2^).

On hMSCs, red PBM caused a moderate but significant increase of cell proliferation, in accordance with previous observations on murine MSCs [11] and on rat MSCs [39,40,41]. These results are also in line with findings by Wang et al. [19] showing the capability of red PBM (660 nm, 3 J/cm^2^) to stimulate proliferation of human adipose-derived stem cells (hASCs), which share many features with bone marrow-derived MSCs [42,43].

This cell proliferative response could be of clinical interest taking into account that proliferation is a crucial event occurring after endogenous MSCs—from the periosteum, endosteum, bone marrow or systemic circulation—are recruited to the bone injury site before they differentiate in bone-forming osteoblasts and/or release a broad range of factors with multiple effects on the bone regenerating microenvironment [5]. Similar to osteoblasts, hMSCs subjected to PBM with 635 nm displayed an increase of the focal adhesion-localized vinculin, are shown to play a role in the promotion of their osteogenic differentiation [44]. Moreover, 635 nm, as compared to 405 nm, upregulated in these cells the expression of Runx-2, the master transcription factor inducing their commitment to the osteogenic lineage [45], and of ALP, as well as increased deposition of mineralized bone-like nodule structures. These findings are in accordance with the report by Wang and coworkers [16] demonstrating the promotion of osteogenic differentiation of hASCs by red PBM (660 nm, 3 J/cm^2^); however, this research observed that blue (420 nm) and green (540 nm) wavelengths have most pronounced effect on hASCs differentiation process when compared to red ones.

Interestingly, we demonstrated that the effects of the red PMB on osteoblasts are mediated by Akt pathway based on: (i) increased expression levels of Akt and of its activated phosphorylated form, p-Akt, in the cells irradiated with 635 nm as compared to untreated cells; and (ii) abolishment of the effects induced by 635 nm (i.e., vinculin upregulation and osteogenic differentiation) on osteoblasts following the blockade of Akt by triciribine. These findings are consistent with previous reports showing the pivotal role of this signaling pathway in focal adhesion dynamics in different cell types [46,47,48,49], as well as in the bone cell survival, functionality and differentiation [50,51,52]. Our data are also in accordance with previous studies showing Akt signaling as molecular target of different PBM treatment in different cell types [53,54,55,56]. In addition, the findings showing the decrease of ROS levels when red light was applied to osteoblasts in the presence of triciribine, as compared to the absence of Akt inhibitor, may suggest that Akt signaling mediate the 635 nm-induced osteoblast responses, possibly via a positive regulation of ROS production as occurring in others cell systems [57,58].

The observed slight but significant augmentation of osteoblast ROS levels induced by red light, likely resulting from the classical and well-known laser induced changes in the activity of mitochondrial photoacceptors, such as cytochromes and flavins of the respiratory chain [9], is in accordance with previous observation in other cells [19]. These results may suggest that an upregulation of ROS encourages osteoblast differentiation as reported by other researchers [59,60]. However, the role of ROS in osteoblasts is still controversial. Indeed, other reports showed that a down-regulation of ROS or decreased mitochondrial stress are associated with osteoblast differentiation [61,62]. Therefore, the correlation of ROS levels and osteoblast differentiation in our cell system, their involvement in laser-induced response as well as their link with Akt pathway, needs to be more deeply investigated and we are performing further targeted experiments in this direction.

The modes by which laser irradiation could activate Akt signaling remain to be fully elucidated. Taking in consideration the multi-faceted cross-talk between Akt-mediated pathway and ROS metabolism, a ROS-dependent Akt activation could not be excluded [58]. Akt activation in osteoblasts after 635 nm laser irradiation could be also correlated with laser-induced increase of intracellular ATP content [9,19,55], according to the results of previous reports [63,64]. The red light-induced ATP increase could also justify the slight but significant augmentation of vinculin-rich focal adhesion aggregates, as well as of OPN expression, in osteoblasts irradiated in the presence of the Akt inhibitor, as compared to osteoblasts treated with the inhibitor but not irradiated. This hypothesis is in accordance with the study by Tan et al. [65] which shows that the stimulation of primary osteoblasts with ATP induces transient vinculin clustering at sites of high intracellular traction force, and with the recent report by Gao et al. [62] demonstrating an increase of ATP levels and of mitochondrial complex activities during osteoblast differentiation. Moreover, we have recently demonstrated that low-intensity irradiation with 635 was able to modulate, in fibroblastic cells, the functionality of Transient Receptor Potential Canonical (TRPC)-1 membrane calcium channels [32], whose involvement in Akt signaling activation has been reported in different cell types [66,67,68]. Our research group has also shown that osteoblasts express TRPC1 channels, whose activity positively affects vinculin clustering and cell differentiation, and that such channel expression and functionality are modulated by low level Nd:Yag laser irradiation [69]. Therefore, it can be speculated that 635 nm-induced activation of Akt in osteoblasts could be mediated by Ca^2+^ influx through these plasmamembrane channels.

The involvement of Akt in mediating the MSC responses to PBM with 635 nm could be also postulated, in particular taking into consideration the results of Wu and coworkers [41] on MSCs subjected to low-level laser irradiation with 635 nm, demonstrating the up-regulation of the gene expression levels of Akt1 and of its upstream regulator phosphatidylinositol 3-kinase (PI3K). In addition, based on our previous results showing that 635 nm in bone marrow-MSCs stimulates Notch-1 pathway activation via Kir channels [11] and the reported cross-talk between Notch and Akt signaling pathway in human bone marrow-MSCs [70], a diode laser-induced Notch-dependent Akt activation could be also postulated. However, experiments are ongoing in our lab to verify these hypotheses in our experimental cell system.

Taking into consideration the results showing the increase of vinculin expression aggregation and calcium deposits in both osteoblasts and hMSCs and of Runx-2 expression in osteoblasts after irradiation with 808 nm, our study seems also to suggest that PBM with 808 nm could positively influence osteogenic differentiation of these two types of progenitor cells, as proposed by other research groups [71,72,73,74,75,76]. Along these lines, it has also been reported that PBM using NIR light (810 nm and 980 nm) encourages osteoblastic differentiation of hASCs [16,77].

However, in our experimental model, 808 nm did not up-regulated, in osteoblasts, neither the expression of OPN nor ALP, which is in agreement with the research by Emes et al. [78] on rat calvarial osteoblast-like cells, by Bölükbaşı Ateş et al. [30] on human CRL-11372 osteoblast like-cells and by Pagin et al. [79] on pre-osteoblasts MC3TR; moreover, such treatment, in hMSCs, reduced the expression of Runx-2 and did not augment that of ALP in accordance to Bouvet-Gerbettaz et al. [80].

The uneven trend of differentiation markers makes it difficult to assert with certainty that this kind of NIR phototreatment, performed with the indicated energy density and time parameters, is able to promote osteoblast and hMSC osteogenic differentiation and stresses the need for further investigation.

A very important issue that we must take into consideration is the possibility of the occurrence of a biphasic-dose osteoblast and hMSC response to red/NIR light with a peak dose response, as observed in other cell types [19,31,77,81]. This phenomenon could help to explain discordant results among different studies. In other words, this means that when light is delivered at lower doses it could stimulate cellular effects inducing an upregulation of the mediators of cell-signaling pathways responsible for the biostimulatory effects of the light; by contrast, when light is delivered at high doses, the biological stimulation may disappear and even be replaced by inhibition. Therefore, different red/NIR light doses are worth testing in our experimental system to verify the occurrence of the biphasic response and to better understand this phenomenon. This appears necessary to define the best PBM parameters and consequently the best effects on osteoblast progenitor functionality and to translate this information into clinical protocols.

Finally, it is worth noting how many studies reported that a wide range of microbial species including Gram+, Gram−, and yeast, playing a critical role in the pathogenesis of periodontal and peri-implant disease or causing infections during/after orthopedic arthroplasty surgery, are selectively inactivated by 405 nm light [33,35]. The results of this study, showing no significant cell responses induced by violet-blue PBM, may be of clinical interest, suggesting the ability of 405 nm to preserve host cell functionality even during a decontamination treatment; in addition, our findings may suggest that the combination of different wavelengths, each with specific and different targets, may represent the most effective treatment aimed to improve clinical outcomes.

## 4. Materials and Methods

### 4.1. Cell Culture and Treatment

*Human osteoblasts-like Saos-2 cells* obtained from American Type Culture Collection (ATCC Manassas, VA, USA) were cultured in proliferation medium (PM) containing F12-Coon’s modification medium (Sigma, Milan, Italy), 10% fetal bovine serum (FBS; Sigma), 1% l-glutamine and 100 U/mL penicillin-streptomycin (Sigma). To stimulate osteogenic cell differentiation, the cells were cultured for different times (7 and 18 days) in differentiation osteogenic medium (DM, F12-Coon’s modification medium plus 10% FBS, 1% l-glutamine and 100 U/mL penicillin-streptomycin, 100 μg/mL ascorbic acid, 10 mM β-glycerophosphate, 10 nM dexamethasone; Sigma). DM was replaced every 3–4 days. In some experiments to evaluate the involvement of Akt signaling in the osteoblast responses induced by PBM with 635 nm, the cells were cultured for 24 h in PM or for 7 days in DM, in the presence of a specific Akt inhibitor, triciribine (2 μM (T2), Sigma) [82]. Triciribine stock solution was prepared in dimethyl sulfoxide (DMSO); the DMSO final concentration in the culture medium was 0.1% and the same dose of DMSO was added to corresponding controls.

*Human bone marrow-derived MSCs (hMSCs)* were isolated from iliac crest and aspirates of normal donors, cultured and immunophenotypically and morphologically characterized as previously reported [83]. All donors gave their informed consent before being included in the study which. was conducted according to the Declaration of Helsinki and approved by the local Ethics Committee of University of Florence, Italy (# Prot. 23/2007). hMSCs were cultured in PM containing Dulbecco Eagle modified medium (DMEM) plus GlutaMAX™ (Sigma), 20% FBS, 2 mM l-glutamine and 100 U/mL penicillin and 100 μg/mL streptomycin (Sigma). Osteogenic cell differentiation was induced by culturing the cells for 7 and 18 days in osteogenic DM where F12-Coon’s modification medium was substituted by DMEM+ GlutaMAX™. DM was replaced every 3–4 days.

PBM treatments were performed on cells cultured on wells of 6-wells/plates (well diameter: 30 mm) or of 24-wells/plates (well diameter: 18 mm) or on glass cover-slips (20 × 20 mm, put on the bottom of a well of 6 wells/plates; round cover-slips put on the bottom of wells of 24 wells/plates)).

### 4.2. PBM Treatments

PBM treatments were carried out by using two diode lasers (Dental Laser System 4 × 4^TM^, General Project Ltd., Montespertoli, Florence, Italy) one emitting at λ = 635 ± 5 nm and the other at λ = 808 ± 10 nm (GaAlAs laser) and a LED emitting at λ = 405 ± 5 nm (Dental Laser System 4 × 4^TM^), all operating in continuous wave in non-contact mode. Laser/LED specifications are indicated in Table 1. During the treatment, the temperature was recorded by a thermal probe included in the laser system console and monitored by a thermal camera (Ti9, Fluke Corp., Everett, WA, USA) showing a thermal map of the irradiated area (data not shown). The temperature remained unchanged during PBM treatments, as previously reported [84,85]. For the duration of PBM treatments, the cover cell culture plate was removed, and all the procedures were carried out under “clean bench” conditions to prevent contamination. To minimize light reflection a black background in the irradiation areas was positioned.

### 4.3. Cell Viability

Cell viability was first evaluated by propidium iodide (PI)/Syto 16 DNA staining test essentially as previously reported [86]. PI-stained nuclei indicated dead cells, whereas Syto 16-stained nuclei the viable ones. In detail, the cells grown on glass cover slips, subjected or not to PBM treatments, were incubated with a mix solution of PI (1:100, Molecular Probes, Eugene, OR, USA) and Syto 16 (1:2000, Molecular Probes) in PBS for 15 min at 37 °C, then washed and fixed in 0.5% buffered paraformaldehyde (PFA) for 10 min at room temperature. The glass coverslips with the stained cells were observed under a confocal Leica TCS SP5 microscope (Leica Microsystems, Mannheim, Germany) equipped with a HeNe/Ar laser source for fluorescence measurements and with differential interference contrast (DIC) optics. Observations were performed using a Leica Plan Apo 63×/1.43NA oil immersion objective. Series of optical sections (1024 × 1024 pixels each; pixel size 204.3 nm) 0.4 μm in thickness were taken through the depth of the cells at intervals of 0.4 μm. Images were then projected onto a single “extended focus” image. For each cell preparation (at least three independent experiments, performed in triplicate) the number of Syto 16 positive viable cells was evaluated in at least 10 random 200 × 200 μm^2^ microscopic fields (63× objective) and reported as percentage of total cells.

Cell viability was also evaluated by MTS assay (Promega Corp., Madison, WI, USA), as previously reported with minor modifications [32]. Briefly, the cells were seeded in wells of 24-wells/plate (3 × 10^4^ Saos-2 cells/well, 1.5 × 10^4^ hMSCs/well; well diameter: 18 mm) and cultured in their specific phenol red-free PM for 24 h before being subjected to the three different PBM treatments; after next 24 h the cells were incubated with MTS test solution (40 µL) in fresh phenol red-free PM (200 µL). The optical density (OD) of soluble formazan deriving the reduction of tetrazolium by mitochondrial enzymes of viable cells, was measured after 4 h, using a multi-well scanning spectrophotometer (ELISA reader; Amersham, Pharmacia Biotech, Cambridge, UK) at a wavelength of 492 nm.

### 4.4. Morphological Analyses

#### 4.4.1. Phase Contrast Microscopy

Cell morphology was daily monitored under an inverted phase contrast microscope (Nikon Diaphot 300, Nikon, Tokyo, Japan).

#### 4.4.2. Confocal Laser Scanning Microscopy

The cells cultured on glass cover-slips, subjected or not to PBM treatments, were fixed with 0.5% PFA for 10 min at room temperature, permeabilized with 0.5% Triton X in PBS for 10 min and blocked with 0.5% bovine serum albumin (BSA; Sigma) and 3% glycerol in PBS for 20 min before being incubated for 1 h at room temperature with mouse monoclonal anti-vinculin (1:100; Sigma) or overnight at 4 °C with the rabbit polyclonal anti-Ki67 (1:100; Santa Cruz Biotechnology, Santa Cruz, CA, USA) or rabbit polyclonal anti-osteopontin (1:50; Santa Cruz) or rabbit monoclonal anti-p-Akt (1:100; Cell Signaling Technology, Signaling Technology, Danvers, MA, USA) primary antibodies. The immunoreactions were revealed by incubation with specific anti-mouse or anti-rabbit Alexa Fluor 488- or 568-conjugated IgG (1:200; Molecular Probes) for 1 h at room temperature. In some experiments, cells were stained with Alexa 488-labeled phalloidin (1:40; Molecular Probes) to detect actin filament organization or with PI (1:30; Molecular Probes) to label nuclei. Negative controls were performed by substituting the primary antibodies with non-immune serum; cross-reactivity of the secondary antibodies was evaluated by omitting the primary antibodies. The glass coverslips with the immunolabelled cells were observed under the confocal Leica TCS SP5 microscope (Leica Microsystems). The observations were performed as described above in the Section 4.3. The number of Ki67 or p-Akt positive nuclei were counted in 10 random 200 × 200 μm^2^ microscopic fields (63× objective) in each cell preparation and expressed as percentage of the total cell nuclei. The analysis was performed by two different operators in at least three different cell preparations for each experimental condition; experiments were performed in triplicate. Densitometric analyses of vinculin and OPN fluorescent signal intensity were performed on digitized images by utilizing ImageJ software (Available online: http://rsbweb.nih.gov/ij) in 20 regions of interest (100 μm^2^) for each confocal stack (at least 10).

### 4.5. Reverse Transcription Polymerase Chain Reaction (RT-PCR)

The expression levels of Runx-2 and ALP mRNA were determined by RT-PCR. Briefly total RNA extraction was performed by using TRIzol Reagent (Invitrogen, Grand Island, NY, USA), according to the manufacturer’s instructions, from cells cultured on wells of 6-wells/plates, subjected or not to PBM treatments. One µg of total RNA was reverse transcribed and amplified with SuperScript One-Step RT-PCR System (Invitrogen, Grand Island, NY, USA). After cDNA synthesis at 55 °C for 30 min, the samples were pre-denatured at 94 °C for 2 min and then subjected to 40 cycles of PCR performed at 94 °C for 15 s, alternating with 57 °C for 30 s (Runx-2 and ALP) or 55 °C for 30 s (β-actin) and 72 °C for 1 min; the final extension step was performed at 72 °C for 5 min.

The following human gene-specific primers were used: Runx-2 (NM_001015051.3), forward 5′-CT TCT GTG GCA ACT TT-3′ and reverse 5′-AAG GAC CAG AGA ACA AGG GG-3′; ALP (X55958.1), forward 5′-GGG CAA CTT CCA GAC CAT TG-3′ and reverse 5′-GAG TAC CAG TTG CGG TTC AC-3′; β-actin (NM_001101.3), forward 5′-AAA CTG GAA CGG TGA AGG TG-3′ and reverse 5′-CTG TGT GGA CTT GGG AGA GG-3′. β-actin mRNA was used as internal standard. Blank controls, consisting of no template (water), were performed in each run. PCR products were separated by electrophoresis on a 1.8% agarose gel and the ethidium bromide-stained bands were quantified by densitometric analysis by using ImageJ software (Available online: http://rsbweb.nih.gov/ij). β-actin normalization was performed for each result.

### 4.6. Fluorescent Mineralized Nodules Assay

Fluorescent analysis of mineralized bone-like nodule structures (Ca^2+^ deposits) formation was performed by using Fluorescent Osteolmage™ Mineralization assay (Lonza Walkersville, Inc., Walkersville, MD, USA) essentially as previously reported [83]. The fluorescent Osteolmage™ staining reagent can bind the hydroxyapatite portion of the bone-like nodules peculiar of the mature osteoblastic cells. Briefly the cells on glass cover-slips subjected or not to PBM treatments, were cultured for 18 days in their specific osteogenic DM and the fixed with 0.5% buffered PFA. After that the cells were incubated with the fluorescent Osteolmage™ staining reagent for 30 min at room temperature in the dark, washed and observed under confocal Leica TCS SP5 microscope. The observations were performed as reported in the Section 4.3. Densitometric analysis of the intensity of mineralized nodules (Ca^2+^ deposits) fluorescent signal was performed on digitized images using ImageJ software (Available online: http://rsbweb.nih.gov/ij) in 20 regions of interest (ROI) of 100 μm^2^ for each confocal stack (at least 10).

### 4.7. Western Blotting

Total protein extraction from human osteoblasts-like Saos-2 cells cultured on wells of 6-wells/plates and protein quantification were performed as reported previously [82]. Forty micrograms of total proteins were electrophoresed on NuPAGE^®^ 4–12% Bis-Tris Gel (Invitrogen, Life Technologies, Grand Island, NY, USA; 200 V, 40 min) and blotted onto polyvinylidene difluoride (PVDF) membranes (Invitrogen, Life Technologies; 30 V, 1 h). The membranes were incubated overnight at 4 °C with rabbit monoclonal anti-Akt (1:1000; Cell Signaling Technology), rabbit monoclonal anti-p-Akt (1:1000; Cell Signaling Technology) and rabbit polyclonal anti α-tubulin (1:1000; Merck, Milan, Italy) antibodies. Specific bands were detected with Western Breeze^®^Chromogenic Immunodetection kit (Invitrogen, Life Technologies, Grand Island, NY, USA). Densitometric analysis of the bands was performed using ImageJ software (Available online: http://rsbweb.nih.gov/ij) and the values normalized to α-tubulin.

### 4.8. ROS Generation Detection

The intracellular levels of ROS were measured using the fluorescent probe 2′,7′–dichlorofluorescin diacetate, acetyl ester (CM-H_2_ DCFDA; Sigma-Aldrich). Human osteoblasts-like Saos-2 cells plated at a density of 90,000 cells on well on 24-well plates or on round glass cover-slip (put on the bottom of wells of 24 wells/plates), were subjected to red PBM in the presence or absence of Akt inhibitor, triciribine (2 μM). After PBM treatment the cells were incubated with 5 μM CM-H_2_ DCFDA in PBS solution for 1 h in dark at 37 °C. The fluorescence values at 538 nm were detected by Fluoroskan Ascent FL (Thermo-Fisher, Illkinch, France). Meanwhile, the cells on glass coverslips were fixed with 0.5% PFA for 10 min at room temperature and processed to be observed under confocal microscope. Densitometric analysis of the intensity of CM-H_2_ DCFDA green fluorescent signal was performed on digitized images using ImageJ software (Available online: http://rsbweb.nih.gov/ij) in 20 regions of interest (ROI) of 100 μm^2^ for each confocal stack (at least 10).

### 4.9. Statistical Analysis

Data are reported as mean ± standard error of the mean (S.E.M.) of at least three independent experiments performed in triplicates. Statistical comparison of differences among the experimental groups was performed using one-way ANOVA with post-hoc Tukey HSD. A *p* value < 0.05 was considered significant. Calculations were done using the GraphPad Prism 4.0 statistical software (GraphPad, San Diego, CA, USA).

## Figures and Tables

**Table 1:** Light sources and irradiation parameters.

Diode Laser/LED Beam Characteristics			
	*Diode Laser 635*	GaAlAs *Diode Laser 808*	*LED 405*
Wavelength (λ)	635 ± 5 nm	808 ± 10 nm	405 ± 5 nm
Irradiation mode	continuous wave	continuous wave	continuous wave
Handpiece type	Focalized zoom handpiece	Fiber NA = 0.22	Light pipe glass
Applicator Diameter		0.6 mm	10 mm
Beam Power	89 mW	400 mW	500 mW
Surface treatment data			
Treatment mode	Non-contact	Non-contact	Non-contact
Distance from the target	30 mm	66 mm	11 mm
Target diameter/surface area	30 mm/706.9 mm^2^ 18 mm/273 mm^2^ 18 mm/273 mm^2^	30 mm/706.9 mm^2^ 18 mm/273 mm^2^	30 mm/706.9 mm^2^ 18 mm/273 mm^2^
Exposition time	30 s	30 s	30 s
Power density	12.59 mW/cm^2^	12.59 mW/cm^2^	12.59 mW/cm^2^
Energy density at target level	0.378 J/cm^2^	0.378 J/cm^2^	0.378 J/cm^2^
